# Combining COPD with Clinical, Pathological and Demographic Information Refines Prognosis and Treatment Response Prediction of Non-Small Cell Lung Cancer

**DOI:** 10.1371/journal.pone.0100994

**Published:** 2014-06-26

**Authors:** Joseph Putila, Nancy Lan Guo

**Affiliations:** Department of Environmental and Occupational Health Sciences, School of Public Health, Mary Babb Randolph Cancer Center, West Virginia University, Morgantown, West Virginia, United States of America; Univesity of Texas Southwestern Medical Center at Dallas, United States of America

## Abstract

**Background:**

Accurate assessment of a patient’s risk of recurrence and treatment response is an important prerequisite of personalized therapy in lung cancer. This study extends a previously described non-small cell lung cancer prognostic model by the addition of chemotherapy and co-morbidities through the use of linked SEER-Medicare data.

**Methodology/Principal Findings:**

Data on 34,203 lung adenocarcinoma and 26,967 squamous cell lung carcinoma patients were used to determine the contribution of Chronic Obstructive Pulmonary Disease (COPD) to prognostication in 30 treatment combinations. A Cox model including COPD was estimated on 1,000 bootstrap samples, with the resulting model assessed on ROC, Brier Score, Harrell’s C, and Nagelkerke’s R^2^ metrics in order to evaluate improvements in prognostication over a model without COPD. The addition of COPD to the model incorporating cancer stage, age, gender, race, and tumor grade was shown to improve prognostication in multiple patient groups. For lung adenocarcinoma patients, there was an improvement on the prognostication in the overall patient population and in patients without receiving chemotherapy, including those receiving surgery only. For squamous cell carcinoma, an improvement on prognostication was seen in both the overall patient population and in patients receiving multiple types of chemotherapy. COPD condition was able to stratify patients receiving the same treatments into significantly (log-rank *p*<0.05) different prognostic groups, independent of cancer stage.

**Conclusion/Significance:**

Combining patient information on COPD, cancer stage, age, gender, race, and tumor grade could improve prognostication and prediction of treatment response in individual non-small cell lung cancer patients. This model enables refined prognosis and estimation of clinical outcome of comprehensive treatment regimens, providing a useful tool for personalized clinical decision-making.

## Introduction

Lung cancer is the leading cause of cancer-related deaths in industrialized countries [1]. Non-small cell lung cancer (NSCLC) accounts for about 80% of lung cancer cases. Major histology of NSCLC includes lung adenocarcinoma and squamous cell lung carcinoma. Tumor recurrence and metastasis is the major treatment failure (i.e., death) of lung cancer. In the current practice, surgical resection is the major treatment option for stage I NSCLC patients. Nevertheless, up to 50% of stage I NSCLC patients will develop and die from tumor recurrence within five years following the surgery [2], [3]. It is therefore important to select early stage NSCLC patients for more aggressive treatment. On the other hand, patients with positive lymph node involvements are recommended to receive adjuvant chemotherapy. However, the benefits of chemotherapy on NSCLC are not convincing [4]. Recent meta-analysis of 12 randomized trials showed that the use of combination chemotherapy had significantly better clinical outcome than single agent chemotherapy [5]. Currently, a clinical decision scheme is needed to recommend which chemotherapy and treatment course would be optimal for a particular patient.

One of the most important factors influencing the survival of patients with lung and bronchus cancers is the selection of an appropriate course of therapy based on an accurate assessment of patient risk. However, the selection of a course of therapy is currently based largely on cancer stage alone despite the contribution of other factors to patient survival. In particular, comorbidities such as COPD can have a significant effect on long-term survival due to varying treatment candidacy, increased complication rate, or decreased treatment efficacy [6]–[8], and the prevalence of COPD is elevated in lung cancer patients independent of age, gender, and smoking history [9]. A major impediment is determining the contribution of each of these factors, including COPD, cancer stage, tumor grade, age, gender, race and histology, in a comprehensive prognostic model. It would be clinically useful to develop such a model to assess individual patient treatment outcome in comprehensive therapeutic regimens, including surgery, radiation, and multiple chemotherapeutic agents, by using large-scale patient medical records.

Comorbidities which affect lung function, such as COPD, are likely to influence post-operative survival independent of their effects on surgical candidacy due to the possibility of decreased lung function. The Forced Expiratory Volume in 1 second (FEV_1_) of a patient, a potential indicator of COPD, was found to be a significant prognostic factor in a model controlling for other clinical variables such as nodal status [10], [11]. Although COPD and lung cancer are often seen together in patients due to their shared association with smoking behavior, neither condition is a clinical end-point for the other. Therefore, it is important to investigate whether COPD is a potential prognostic factor of lung cancer and how to incorporate it in the treatment selection.

Previously, we developed a prognostic model utilizing similar data derived from the Surveillance Epidemiology and End-Results (SEER) cancer registry initiative [12]. The previous approach utilized clinical, pathological, and demographic variables in a single model to achieve superior prognostication over a similar model using cancer stage alone. This model was however limited by the data in that the use of specific chemotherapies and the presence of comorbidities could not be determined. This study extends the previous analysis by utilizing patient medical records from the linked SEER-Medicare database. These additional data allow for the role of COPD in prognostication to be determined across combinations of surgical, radiological, and chemotherapeutic treatments [13]–[15]. In this study, we sought to 1) develop a comprehensive lung cancer prognostic model by incorporating information of COPD, age, gender, race, tumor grade, cancer stage, AJCC staging edition, and histology; 2) estimate 2-year and 5-year survival probability of patients receiving surgery, radiation, and chemotherapy, including Platinum-based, Platinum/Taxane, and Carboplatin/Paclitaxel/Avastin; 3) develop an online NSCLC prognostic tool for individualized clinical decision-making.

## Patients and Methods

Patient data was obtained from the linked SEER-Medicare database, a combination of population-based registry data and billing histories for patients covered by Medicare [16]. In short, data from participating SEER registries were linked with Medicare data through the use of social security number, census tract, age, and other identifying variables [15]. The resulting data contain information on treatments administered, co-morbidities present in the patient, clinical presentation, survival, and demographics. Criteria for inclusion in this analysis were a diagnosis of lung or bronchus cancer between 1991 and 2005, and complete information on age, race, gender, tumor grade and stage, as well as valid follow-up and billing history. This set of patients was further refined to only include those with tumors broadly classifiable as either squamous cell carcinoma or adenocarcinoma. Cases reported solely from autopsy or death certificate were excluded. In total, 34,203 patients with lung adenocarcinomas and 26,967 patients with squamous cell lung carcinomas met these criteria (Table 1).

**Table 1:** Distribution of demographic and clinical characteristics of patients diagnosed with adenocarcinoma or squamous cell carcinoma in the original AJCC 3^rd^ and 6^th^ staging editions and recoded 7^th^ edition.

Patient Information	Adenocarcinoma			Squamous Cell Carcinoma		
	AJCC 3^rd^	AJCC 6^th^	AJCC 7^th^	AJCC 3^rd^	AJCC 6^th^	AJCC 7^th^
**Stage**						
***1***	9,510 (35.6%)	3,127 (42%)	2,371 (33.3%)	7,110 (33%)	1,968 (36.5%)	1,466 (28.6%)
***2***	1,475 (5.5%)	580 (7.8%)	1,400 (19.7%)	1,272 (5.9%)	574 (10.6%)	958 (18.7%)
***3a***	2,610 (9.8%)	661 (8.9%)	1,145 (16.1%)	3,074 (14.2%)	650 (12%)	1,114 (21.7%)
***3b***	4,638 (17.3%)	919 (12.3%)	438 (6.2%)	4,928 (22.8%)	907 (16.8%)	496 (9.7%)
***4***	8,516 (31.8%)	2,167 (29.1%)	1,762 (24.8%)	5,188 (24.0%)	1296 (24%)	1,089 (21.3%)
**Grade**						
***1***	3,436 (12.8%)	1,189 (16%)	1,143 (16%)	1,000 (4.6%)	191 (3.5%)	180 (3.5%)
***2***	8,780 (32.8%)	2,892 (38.8%)	2,800 (39.3%)	8,384 (38.9%)	2,284 (42.3%)	2,182 (42.6%)
***3***	13,885 (51.9%)	3,235 (43.4%)	3,044 (42.8%)	11,713 (54.3%)	2,850 (52.8%)	2,694 (52.6%)
***4***	648 (2.4%)	138 (1.9%)	129 (1.8%)	475 (2.2%)	70 (1.3%)	67 (1.3%)
**Age, Mean(SD)**	73.4 (7.2)	74.0 (7.6)	73.9 (7.6)	73.5 (6.9)	74.1 (7.4)	74.2 (7.3)
**Race**						
***White***	23,218 (86.8%)	6,515 (87.4%)	6,219 (87.4%)	18,456 (85.6%)	4,727 (87.6%)	4,490 (87.6%)
***Black***	1,945 (7.3%)	531 (7.1%)	508 (7.1%)	2,232 (10.3%)	486 (9%)	459 (9.0%)
***Asian/Pac. Islander***	1,586 (5.9%)	408 (5.5%)	389 (5.5%)	884 (4.1%)	182 (3.4%)	174 (3.4%)
**Sex**						
***Male***	13,678 (51.1%)	3,556 (47.7%)	3,372 (47.4%)	14,111 (65.4%)	3,370 (62.5%)	3,186 (62.3%)
***Female***	13,071 (48.9%)	3,898 (52.3%)	3,744 (52.6%)	7,461 (34.6%)	2,025 (37.5%)	1,937 (37.7%)
**+COPD Diagnosis**	7,948 (29.7%)	2,647 (35.5%)	2,538 (35.7%)	8,521 (39.5%)	2,568 (47.6%)	2,431 (47.6%)

The administration of chemotherapy was determined using Healthcare Common Procedure Coding System (HCPCS) codes in combination with International Classification of Diseases (ICD) codes. For each patient, all records were searched for entries with an HCPCS code indicating that chemotherapy was administered. These entries were then cross referenced with the ICD primary and secondary diagnosis codes for that entry to ensure that the agent was being administered for the treatment of lung or bronchus cancers. Four specific agents, cisplatin, carboplatin, docetaxel, and paclitaxel were considered in addition to a broad group covering a variety of chemotherapeutic agents.

Patients were also analyzed according to variable administration of curative surgery and radiological treatment. Five groups were formed on the basis of surgical and radiological treatments; a group containing all patients regardless of treatment, a group for patients having surgery without radiation, another for those having radiation without surgery, a group with both surgery and radiation, a group of patients without any treatment listed. Surgical and radiological treatment group assignments were based on the presence of any curative treatment of that type in the SEER portion of the data.

Comorbidities were measured as components of a version of the Charlson Comorbidity Index (CCI) adapted for use with administrative data [17]–[19]. Comorbidities were determined using code available from the NCI designed specifically for this SEER-Medicare dataset (http://healthservices.cancer.gov/seermedicare/program/comorbidity.html), under the assumption that the presence of a treatment for a specific disease in the billing history was indicative of its presence in the patient at the time. All claims out to three years prior to the diagnosis with lung cancer were analyzed for specific sets of ICD-9 codes indicative of one of the conditions listed in the CCI. Upon finding a relevant ICD code for any of the 18 conditions included in the index, patients were flagged as being positive for the corresponding condition. The determination of chemotherapy use and co-morbidities was done using *SAS* version 9.2 in the PC environment.

Patient age, race, and gender were identified using information in the SEER portion of the data, with Black and Asian/Pacific Islander patients being compared to Whites as a reference group. Tumor grade was also identified from the SEER portion of the data. Tumor grades 1 and 2 were grouped together and served as a reference group for grades 3 and 4, which were also grouped in the Cox models. Cancer stage was listed in the SEER portion of the data in either the 3^rd^ or 6^th^ Edition of the AJCC staging system. Records coded using the 6^th^ Edition were able to be recoded to the 7^th^ Edition provided that they had complete and valid information on tumor size, extension, nodal involvement, and distant metastases. Cancer stage I was used as a reference group in the Cox models. The number and distribution of patient characteristics is detailed in Table 1.

Cox modeling was used to estimate a proportional hazards model for use in quantitatively assessing patient risk given multiple variables. Two separate models were estimated: a Full model containing information on patient age, race, gender, histology, tumor grade and cancer stage as developed in our previous study [12], and a COPD model containing the same variables with an additional indicator of the presence of COPD. The performance of these two models was compared to evaluate whether the addition of COPD could improve prognostication and prediction of treatment outcome. For both the Full and COPD models, a total of 1,000 Cox proportional-hazard models were estimated using bootstrapped samples equal in size to the original patient cohort. The distributions of coefficients were assessed for normality, and the mean of each coefficient was taken. This set of coefficients formed the final model for each approach.

The models were then assessed on a total of four metrics; area under an ROC curve, Brier Score, Harrell’s c, and Nagelkerke’s R^2^. The ROC measure represents the area under an integrated ROC curve out to 60 months for the original 3^rd^ Edition AJCC staging, and 24 months for the 6^th^ and recoded 7^th^ Edition AJCC staging. The Harrell’s *c* measure is similar to the ROC measure, but takes into account the aspect of time. Nagelkerke’s R^2^ is a generalized form of the coefficient of determination (R^2^) suitable for survival models. The ROC, *c*-statistic, and R^2^ measures all range from 0 to 1, with higher scores indicating better performance. The Brier score is a measure of the accuracy of survival predictions, and ranges from 0 to 1 with lower scores being better. Similar to the ROC measure, the Brier score was calculated at 36 months for the original AJCC staging and 24 months for the recoded AJCC staging. These estimations were performed on 1,000 bootstrapped patient cohorts with a two-tailed *t*-test being used to assess significant differences between models on each test [20]. All model estimations and assessments were performed using *R* version x64 2.15.0, with the *survival*, *risksetAUC*, *Design*, *rms*, *pec*, and *Hmisc* packages.

The final models to be used in the online tool were constructed using the coefficients estimated earlier for the models containing cancer stage, tumor grade, histology, age, race/ethnicity, gender, and COPD status. A total of 6 models, one for each AJCC Staging Edition and histology combination, were constructed. Cutoffs for each AJCC Edition and histology group were determined by selecting a cutoff to partition patients into high- or low-risk groups across the range of Hazard Scores in the total patient population and testing the difference in survival between the Full and Stage Only models, respectively, in an iterative manner. The final cutoffs represent points at which the Full model shows the greatest improvement in selecting both high- and low-risk patients in that group. This stratification is represented in Figure S7. The final coefficients and graphical representation of long-term survival as determined by Hazard Score are shown for each model in the results section. To estimate prognosis and treatment response of a new patient, the Hazard Score is estimated using the coefficients for each variable in the corresponding model. A representative image of the web-based application (www.personalizedRx.org) of the final prognostic model is provided in the results.

## Results

### Impact of COPD and other co-morbidities on NSCLC survival

**Figure 1:**
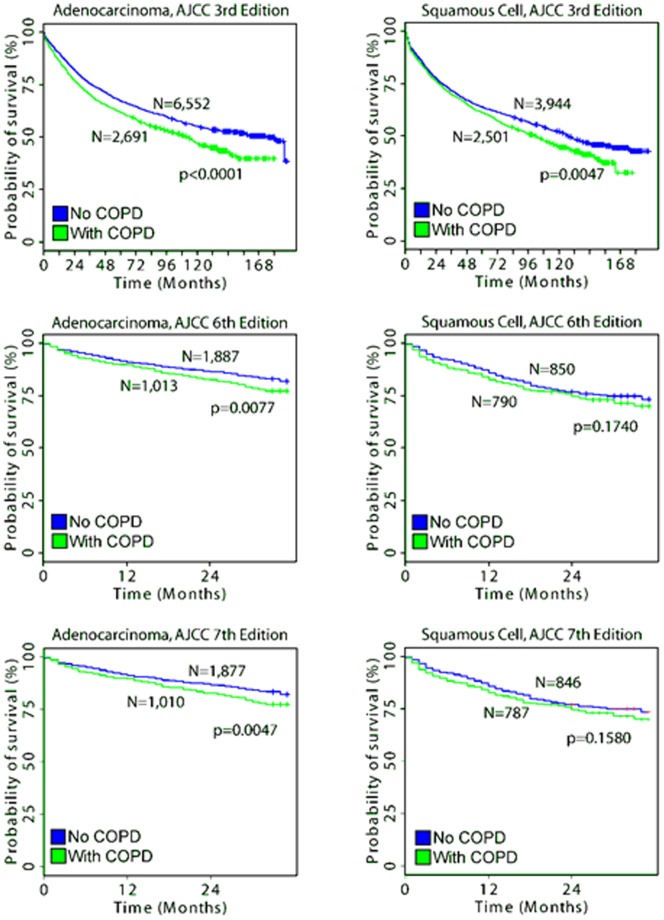
Kaplan-Meier analysis of patients with and without COPD among those treated with surgery alone. Log-rank tests were used to assess the difference in survival probabilities of two groups.

In patients receiving only surgery without radiation or chemotherapy, COPD showed significant association (*p*<0.05; Cox model) with lung cancer disease-specific survival (Table 2). Other co-morbid conditions, including congestive heart failure, peripheral vascular disease, cerebrovascular disease, diabetes with sequelae, and gastrointestinal ulcers, also had a significant (*p*<0.05; Cox model) association with disease-specific survival (Table 2). As an independent prognostic factor, COPD status alone was able to significantly stratify patients into high- and low-risk groups (*p*<0.05) in all lung adenocarcinoma patients and squamous cell lung carcinoma patients diagnosed with the AJCC 3^rd^ edition (Figure 1). In the significant cases, NSCLC patients without COPD showed consistently and significantly better survival when compared to those with COPD across the entire period of post-operative follow-up, indicating that the effects of COPD are manifested in both long- and short-term disease-specific survival (Figure 1). Small sample size in the newer squamous cell carcinoma groups diagnosed with the AJCC 6^th^ Edition and those recoded to the 7^th^ Edition of AJCC staging system may have impeded achieving a statistically significant stratification by COPD. Still, the results indicate COPD patients having shorter disease-specific survival.

**Table 2:** Association of each comorbid condition and NSCLC survival when the patient sample was restricted to patients receiving surgery without radiation or chemotherapy.

	Adenocarcinoma		Squamous Cell	
Condition	Hazard Ratio	p-value	Hazard Ratio	p-value
*Congestive Heart Failure*	1.37	<0.0001*	1.27	0.0003*
*Peripheral Vascular Disease*	1.22	0.0020*	1.03	0.7234
*Cerebrovascular Disease*	1.16	0.0152*	1.06	0.3874
*COPD*	1.24	<0.0001*	1.11	0.0140*
*Diabetes with sequelae*	1.26	0.0186*	1.06	0.5964
*Chronic Renal Failure*	1.18	0.4083	1.57	0.0002*
*Cirrhosis*	0.74	0.3000	1.95	0.0024*
*Gastrointestinal Ulcers*	1.33	0.0193*	1.22	0.1337

* indicates statistical significance at a *P*<0.05 level.

COPD was further established as an independent prognostic factor in the evaluation of stage 1, stage 2 and 3a patient groups receiving specific treatments. Particularly, in stage 1 lung adenocarcinoma and squamous cell lung carcinoma patients receiving surgery without any systemic therapy, those without COPD had significantly (*p*<0.05) better post-surgical survival than those had COPD (Figures S1 to S4). Similar results were observed in stage 2 and 3a NSCLC patients receiving surgery without systemic therapy (Figures S1 to S6). More favorable clinical outcome was also observed in stage 1, stage 2 and 3a patients without COPD compared with those with COPD, who received surgery or radiation with systemic therapy (Figures S1 to S6). These results indicate that COPD is a significant and independent prognostic factor of NSCLC.

### Evaluation of Clinical, Pathological and Demographic Factors in NSCLC prognosis

In order to assess which factors are important determinants in NSCLC prognosis, patient clinical, pathological, and demographic variables were compared in good prognosis (those who survived more than 5 years after diagnosis) and poor prognosis (those who died within 2 years after diagnosis) groups. To reduce the confounding factor of treatment effects on clinical outcome, only patients receiving surgery without radiation or chemotherapy were included in this analysis. The results on patients diagnosed with AJCC 3^rd^ staging system were shown in Table 3. Detailed results on the newer patients diagnosed with AJCC 6^th^ edition and the recoded 7^th^ edition are not included in this manuscript and are available in [21].

**Table 3:** Comparison of patient information in good prognosis (those survived more than 5 years) and poor prognosis (those survived less than 2 years) groups when the patient sample was restricted to patients receiving surgery without radiation or chemotherapy.

	Adenocarcinoma			Squamous		
Variables\Group	Good Prognosis	Poor Prognosis	P-Value	Good Prognosis	Poor Prognosis	P-Value
With COPD (%)	24.2%	34.2%	<0.0001*	34.0%	39.9%	<0.0001*
Cancer stage (mean)	1.17	2.03	<0.0001*	1.26	1.91	<0.0001*
Tumor grade (mean)	2.08	2.46	<0.0001*	2.50	2.53	0.15*
Age (mean)	72.30	73.80	<0.0001*	72.13	73.85	<0.0001*
Gender (Male %)	39.90%	56.50%	<0.0001*	59.08%	68.31%	<0.0001*
Race (API %)	5.70%	4.70%	0.14	3.51%	3.54%	1
Race (Black %)	5.10%	6.40%	0.087	8.68%	8.03%	0.55

These patients were diagnosed with AJCC 3^rd^ staging edition. * indicates statistical significance at a *P*<0.05 level.

Specifically, good prognosis group had significantly lower percentage of patients with COPD (*p*<0.05; *t*-tests). In lung adenocarcinoma patients, 34.2% of patients had COPD in poor prognosis group versus 24.2% with COPD in good prognosis group (*p*<0.0001; *t*-tests). In squamous cell lung cancer patients, 39.9% of patients had COPD in poor prognosis groups versus 34.0% with COPD in good prognosis group (*p*<0.0005; *t*-tests).

Cancer stage was also a significant factor, and poor prognosis group had more advanced cancer stage (*p*<0.0001; *t*-tests) in both lung adenocarcinoma and squamous cell lung carcinima. Tumor grade was a significant (*p*<0.0001; *t*-tests) factor in lung adenocarcinoma, but not in squamous cell lung carcinoma.

Patient age was a significant prognostic factor in lung adenocarcinoma and squamous cell lung carcinoma diagnosed with all AJCC staging systems. The average patient age in lung adenocarcinoma was 73.8 years old in poor prognosis group and 72.3 in good prognosis group (*p*<0.0001; *t*-tests); whereas the average patient age in squamous cell lung carcinoma patients was 73.85 in poor prognosis group and 72.13 in good prognosis group (*p*<0.0001; *t*-tests).

Patient gender was a significant prognostic factor, with more male patients in poor prognosis group than in good prognosis group in both adenocarcinoma and squamous cell lung carcinoma (*p*<0.0001; *t*-tests). The percentage of male lung adenocarcinoma patients was 39.9% in good prognosis and 56.5% in poor prognosis; whereas in squamous cell lung carcinoma, the percentage of male patients was 59.08% in good prognosis group and 68.31% in poor prognosis group.

Patient race was not a significant factor in NSCLC prognosis (Table 3). In good prognosis group of lung adenocarcinoma, there was a higher percentage of API (5.7% vs. 4.7%) and a lower percentage of black (5.1% vs. 6.4%) than that in poor prognosis group, but the difference was not statistically significant. This trend, however, was no observed in squamous cell lung carcinoma.

### Prognostication Improvement with Addition of COPD in Lung Adenocarcinoma

In a previous study, we have shown that integrating patient information, including age, gender, race, cancer stage and tumor grade improves the prognostication accuracy upon the cancer staging system for both lung adenocarcinoma and squamous cell lung carcinoma [12]. In the present study, we have established that COPD is an independent prognostic factor for NSCLC. With the linked SEER-Medicare database, patient treatment information, including specific systemic therapy, could be retrieved. Hence, this study sought to 1) investigate whether the addition of COPD into the comprehensive model could further improve the prognostication of NSCLC; and 2) estimate the clinical outcome of 30 treatment combinations by using this comprehensive model to guide personalized therapy.

In lung adenocarcinoma patients diagnosed with the original AJCC 3^rd^ Edition staging system, the addition of COPD to the comprehensive model resulted in a significant prognostication improvement (*p*<0.05; Harrell’s *c*-statistic) in the total patient group receiving any treatment. Specifically, there was also significant prognostication improvement in patients treated with surgery alone (*p*<0.05; Harrell’s *c*-statistic) and patients without receiving chemotherapy (*p*<0.05; Harrell’s *c*-statistic and ROC measures; more details provided in [21]).

**Figure 2:**
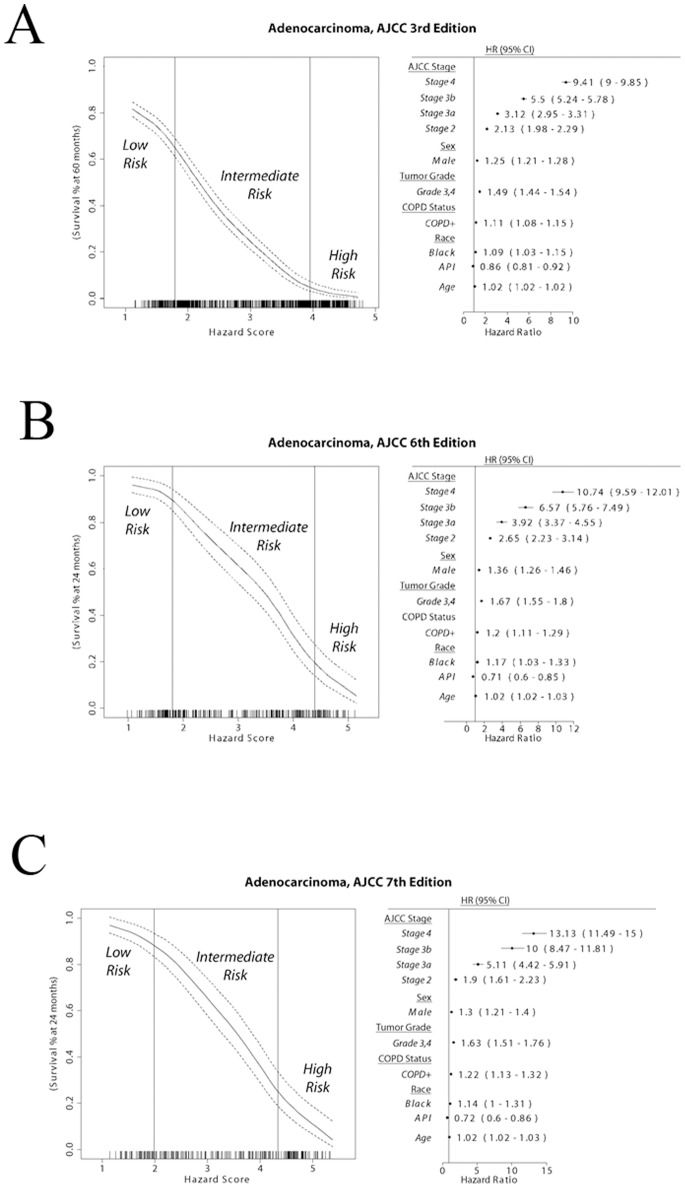
Comprehensive prognostic model for lung adenocarcinoma. Patient survival at 60 months for the total population sample is shown for the range of Hazard Scores (left), with the risk-groups delimited by vertical bars. Model coefficients used to determine the Hazard Score for each patient are shown on the forest plot (right). A: AJCC 3^rd^ Staging Edition; B: AJCC 6^th^ Staging Edition; C: AJCC 7^th^ Staging Edition.

In lung adenocarcinoma patients diagnosed with the AJCC 6^th^ staging system, with the addition of COPD there was a significant prognostication improvement in the total group of patients receiving any treatment (*p* = 0.0480; Harrell’s *c-*statistic). There was an additional prognostication improvement in patients treated with surgery regardless of indication of chemotherapy (*p* = 0.0475; ROC measure). Similar results were observed when patients were recoded to the AJCC 7^th^ Edition staging system. These results indicate that the addition of COPD to the Full model could improve the prognostic accuracy in lung adenocarcinoma patients receiving any treatment, with the greatest prognostication improvement in patients without receiving chemotherapy. The detailed prognostic stratification cutoffs and model coefficients for each staging system can be seen in Figure 2.

### Prognostication Improvement with Addition of COPD in Squamous Cell Lung Carcinoma

There was a significant prognostication improvement (*p* = 0.0239, Brier score) with the addition of COPD to the model in all squamous cell lung carcinoma patients diagnosed with the AJCC 3^rd^ Edition staging scheme. More specifically, when any combination of surgical or radiological treatment was considered regardless of indication of chemotherapy, there was a significant prognostication improvement in the total patient population (*p* = 0.0130; Harrell’s *c*-statistic). This prognostication improvement was also observed in patients treated with any chemotherapy (*p* = 0.0244; Harrell’s *c*-statistic) or with a platinum-based agent (*p* = 0.0125; Harrell’s *c*-statistic). A similar prognostication improvement was seen in patients treated with a platinum-based agent, or with a platinum-based agent and a taxane and any other surgical or radiological treatment (*p*<0.05; Nagelkerke’s R^2^ and ROC measures). The addition of COPD to the model was able to significantly improve prognostication in surgical patients across the range of chemotherapy sub-groups (*p*<0.05; Harrell’s *c*-statistic). These result indicate that the addition of COPD improves the prognostication performance in squamous cell lung cancer overall patient population as well as in multiple chemotherapeutic settings.

**Figure 3:**
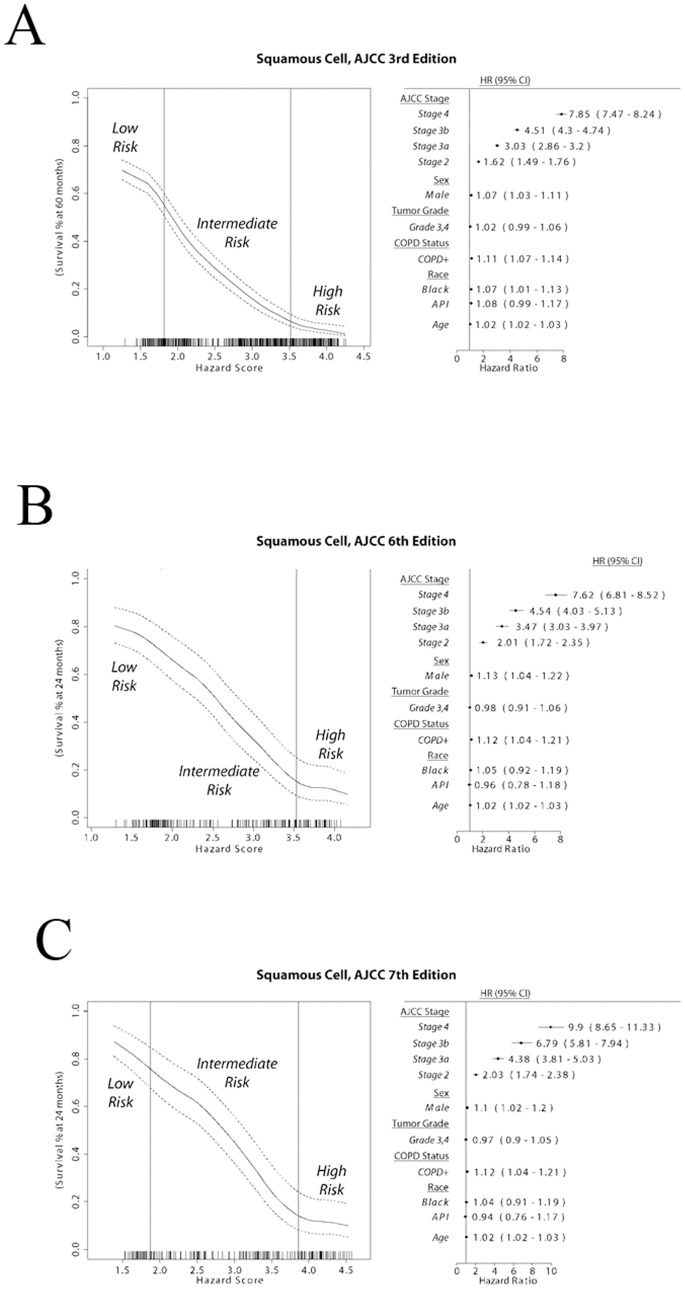
Comprehensive model for squamous cell lung carcinoma. Model coefficients used to determine the Hazard Score for each patient are shown on the forest plot (right). Patient survival at 24 months for the total population sample is shown for the range of Hazard Scores (left), with the risk-groups delimited by vertical bars. A: AJCC 3^rd^ Staging Edition; B: AJCC 6^th^ Staging Edition; C: AJCC 7^th^ Staging Edition.

**Figure 4:**
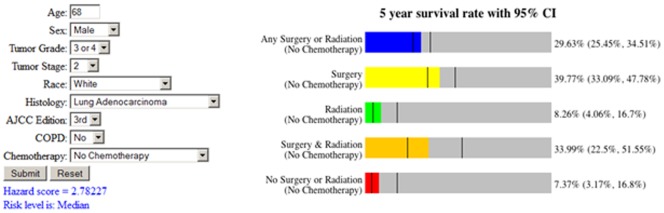
An example of output from the web-based version of the comprehensive prognostic model. Given the patient information submitted by the user (left), the web-based tool will estimate survival for each treatment category using the survival observed for patients of a particular treatment modality and similar Hazard Score (right).

In squamous cell lung carcinoma patients diagnosed with the AJCC 6^th^ staging edition, the addition of COPD into the Full model did not make significant difference in prognostication performance as measured with Nagelkerke’s R^2^, Brier score or ROC measures. There was, surprisingly, a significant degradation in model performance in patients without an indication of treatment (*p* = 0.0089; Harrell’s *c*-statistic). When the patients were recoded using the AJCC 7^th^ Edition criteria, there was a significant prognostication improvement in patients without receiving chemotherapy (*p* = 0.0489; Brier score). These results suggest that the less pronounced prognostication improvement with the addition of COPD in squamous cell lung cancer diagnosed with newer AJCC staging systems might be caused by shorter available follow-up period. The fact that the counter-intuitive results observed in squamous cell lung cancer diagnosed with AJCC 6^th^ staging edition but not with the recoded 7^th^ edition suggests the newest staging system might improve the disease classification. The final prognostic model for squamous cell lung carcinoma patients for each staging system can be seen in Figure 3. An interactive web-based prognostic tool based on the models developed in this study is available at www.personalizedRx.org and an example is provided in Figure 4.

## Discussion

Lung cancer is a dynamic and diverse disease and associated with numerous somatic mutations, deletion and amplification events. The heterogeneous nature of lung cancer makes it a very difficult disease in the clinical managements and it has remained the leading cause of cancer-related deaths for both men and women. Surgical resection is the major treatment option for early stage NSCLC. However, about 35–50% of stage I NSCLC patients will develop tumor recurrence within five years following the surgery [2], [3], and adjuvant chemotherapy of stage II and stage III disease has resulted in very modest survival benefits [22]. It remains a critical issue to recommend appropriate treatment course for an individual patient based on his/her clinical, pathological, demographic and co-morbid conditions, as well as genetic portraits. Using large-scale patient electronic medical records enables the development of a comprehensive prognostic model to estimate treatment response for a particular patient. Such model could potentially be integrated with genetic biomarkers for treatment selection. Our previous study developed a comprehensive prognostic model for NSCLC by using patient information including age, gender, race, cancer stage, tumor grade, and histology [12]. However, this prognostic model did not include co-morbid conditions and particular use of chemotherapy.

Assessment of the effect of COPD in NSCLC prognosis is important due to potential shared origins in the development of lung cancer and COPD [23]–[25] and the role of COPD as a common and important independent risk factor for lung cancer [9]. It is hypothesized that inflammation may initiate or promote tumorigenesis in the lung [23]–[25]. This action is thought to center around the induction of immune dysfunction and the destruction of the extra-cellular matrix [26]. A recent study suggested that the potential role of hypoxic regions of the lung is a possible mechanism for the association between COPD and lung cancer [27]. Both inflammation and hypoxia contribute to the tumor microenvironment and may impact lung cancer prognosis and response to specific treatment. The results from this study demonstrate that NSCLC patients with COPD had significantly worse clinical outcome than those without COPD. In order to account for known confounders such as treatment candidacy and complication rate [6], [28], the effect of COPD was further examined within treatment and cancer stage sub-set. The results show that COPD could significantly stratify patients into different prognostic groups within each specific treatment and cancer stage, indicating that COPD is an independent and significant prognostic factor of NSCLC. The relatively stronger prognostic effect of COPD in squamous cell lung carcinoma compared with lung adenocarcinoma may be indicative of the role of smoking on both survival and the presence of COPD itself, with squamous cell carcinomas being more closely associated with smoking than adenocarcinomas [29]–[31].

The overall co-morbid condition as defined with the CCI index was also significantly associated with NSCLC disease-specific survival in this study (results not shown). Specifically, congestive heart failure, peripheral vascular disease, cerebrovascular disease, diabetes with sequelae, and gastrointestinal ulcers had a significant (*p*<0.05) association with disease-specific survival, respectively. These co-morbid conditions are more likely to derive their prognostic value from confounding or artifactual influences such as socio-economic status or lifestyle differences, instead of molecular mechanistic effects on lung cancer tumor progression. Thus, their inclusion, while potentially beneficial from a statistical standpoint, is imprudent when attempting to model factors directly influencing lung cancer progression and patient survival [32]. In contrast, there was no evidence found in the literature suggesting that the trajectory or treatment of COPD will affect survival estimation in lung cancer patients. For these reasons, COPD alone was added to the prognostic model in lieu of co-morbid conditions.

The SEER data is composed of a geographically and demographically diverse group of patients and thus allows for a more accurate analysis of the prognostic effect of multiple variables. The large size and scope of the data also allow for assessing the accuracy of the model across a wide range of treatments and patient profiles. This is especially relevant when studying the effects of race or comorbidity, factors which have previously been shown to influence survival due to differences in treatments administered [33]. Given the assessment of disease-specific survival stratified by treatment modality, it is highly likely that the effect of COPD represents an additional source of risk to the patient beyond that attributable to reduced candidacy, complication rate, or reduced lung function after treatment. The comprehensive model developed in this study also accounts for recoding of cancer cases to the newest version of the 7^th^ AJCC staging scheme, where possible, and includes both major non-small cell histologies. The prognostic utility of COPD is less pronounced in the AJCC 6^th^ Edition and recoded 7^th^ Edition patients compared with those diagnosed with the AJCC 3^rd^ staging system, possibly because the newer patient groups have both a smaller sample-size and a shorter period of follow-up. It is likely that important manifestations of differences in survival due to COPD may occur beyond the three-year mark, or more recent improvements in the clinical management of COPD cases may alleviate some of the disparity in lung cancer survival, which is beyond the scope of this study.

A limitation of the data is that Medicare coverage is limited to those over the age of 65, with some exceptions, and thus the age distribution of patients included is likely biased toward the upper end of what would be seen in clinical practice. Additionally, the administration of chemotherapeutics or other treatments not covered by Medicare is not recorded in the data, although Medicare covers a significant portion of cancer care in patients who are eligible [34]. As the treatments administered to each patient were non-random, it is also difficult to accurately assess the relative benefit of each type of treatment and thus for this and other reasons the results should not be interpreted as a measure of comparative treatment effectiveness in a randomized clinical trial. In addition, the absence of information on smoking status may also limit the model, as smoking has been shown to influence NSCLC survival independent of co-morbidities [35], [36] and other clinical, pathological, and demographic information [37]. The previous version of the prognostic model without the inclusion of COPD was built on SEER data, and validated with two external patient cohorts [12]. This study extends the previous model with the inclusion of COPD and provides survival estimates for a wide range of treatment modalities including multiple chemotherapeutic agents. While the metrics used to assess model performance indicate that the comprehensive prognostic model presented in this study has a high degree of internal validity, an external validation set was not used because there were no available patient cohorts with sufficient sample size and all the information required in this model.

The administrative data is adequate for assessing co-morbidities and the use of chemotherapeutics [18], [38]. Our results demonstrate that the addition of co-morbid conditions, specifically COPD, to a comprehensive model improves prognostication over similar models without that information. This represents an additional prognostication improvement over the use of cancer stage alone, which has already been validated with statistical rigor [12]. This comprehensive model enables refined prognosis and estimation of clinical outcome of 30 treatment combinations in NSCLC patients, providing a useful tool in personalized clinical decision-making. An online tool based on this model is available at www.personalizedRx.org, which has already been in use in clinics at Mary Babb Randolph Cancer Center. With the advancement of clinical genomics research [39]–[43], this comprehensive prognostic model could be integrated with future genomic biomarkers to predict NSCLC patient treatment response.

## Supporting Information

Figure S1 **Effect of COPD in Adenocarcinoma AJCC 3rd Edition stage and treatment sub-groups.** For each group, a clear and significant difference between the survival curves for patients with and without COPD can be seen, with patients identified as having COPD experiencing significantly poorer survival compared to those without COPD.
(PPTX)

Figure S2 **Effect of COPD in Adenocarcinoma AJCC 6th Edition stage and treatment sub-groups.** For each group, patients without COPD tend to experience longer survival when compared to patients with COPD, although the difference is not significant in some cases shown.
(PPTX)

Figure S3 **Effect of COPD in Adenocarcinoma AJCC 7th Edition stage and treatment sub-groups.** For each group treated without chemotherapy, a clear and significant difference between the survival curves for patients with and without COPD can be seen, with patients identified as having COPD experiencing significantly poorer survival compared to those without the disease. The difference in survival for patients treated with systemic therapy was not significant, but trended toward patients with COPD having poorer survival.
(PPTX)

Figure S4 **Effect of COPD in Squamous Cell AJCC 3rd Edition stage and treatment sub-groups.** For each group, a clear and significant difference between the survival curves for patients with and without COPD can be seen, with patients identified as having COPD experiencing significantly poorer survival compared to those without the disease.
(PPTX)

Figure S5 **Effect of COPD in Squamous Cell AJCC 6th Edition stage and treatment sub-groups.** For the group shown, a clear and significant difference between the survival curves for patients with and without COPD can be seen, with patients identified as having COPD experiencing significantly poorer survival compared to those without the disease.
(PPTX)

Figure S6 **Effect of COPD in Squamous Cell AJCC 7^th^ Edition stage and treatment sub-groups.** For each group, a clear and significant difference between the survival curves for patients with and without COPD can be seen, with patients identified as having COPD experiencing significantly poorer survival compared to those without the disease.
(PPTX)

Figure S7 **Improvement in the Full model using COPD over Stage Alone.** For each Kaplan-Meier plot, the three pairs of lines represent the High, Intermediate, and Low-Risk groups defined for each of the two models shown. The model using only AJCC Stage is shown in orange, while the Full model with COPD status added is shown in blue. For each plot shown, the Full model with COPD status was able to produce a Low-Risk group with better survival and a High-Risk group with poorer survival, with most cases being significant (p<0.05).
(PPTX)
